# Understanding long-term HIV survivorship among African American/Black and Latinx persons living with HIV in the United States: a qualitative exploration through the lens of symbolic violence

**DOI:** 10.1186/s12939-020-01253-w

**Published:** 2020-08-28

**Authors:** Robert Freeman, Marya Gwadz, Leo Wilton, Linda M. Collins, Caroline Dorsen, Robert L. Hawkins, Elizabeth Silverman, Belkis Y. Martinez, Noelle R. Leonard, Amanda Applegate, Sabrina Cluesman

**Affiliations:** 1 Independent Consultant, 205 Clinton Avenue, Brooklyn, NY 11205 USA; 2 Silver School of Social Work, New York University, 1 Washington Square North, New York, NY 10003 USA; 3 Department of Human Development, State University of New York at Binghamton, 4400 Vestal Parkway East, Binghamton, NY 13902 USA; 4 Faculty of Humanities, University of Johannesburg, PO Box 524, Auckland Park, Johannesburg, 2006 South Africa; 5 Department of Human Development and Family Studies, The Methodology Center, The Pennsylvania State University, 435 Health and Human Development Building, University Park, PA 16802 USA; 6 Center for Drug Use and HIV Research, NYU School of Global Public Health, 665 Broadway, 11th Floor, New York, NY 10012 USA; 7 Rory Meyers College of Nursing, New York University, 433 1st Avenue, New York, NY 10010 USA; 8 Edna Bennett Pierce Prevention Research Center, The Pennsylvania State University, 314 Biobehavioral Health Building, University Park, PA 16802 USA

**Keywords:** Symbolic violence, Qualitative, HIV survivorship research, HIV care continuum, Poverty, Race/ethnicity, Disparities, HIV antiretroviral therapy, Adherence, Non-persistence

## Abstract

**Background:**

Persons living with HIV (PLWH) are living longer, although racial/ethnic and socioeconomic status (SES) disparities persist. Yet, little is known about the experience of living with and managing HIV over decades. The present study took a qualitative approach and used the lens of symbolic violence, a type of internalized, non-physical violence manifested in the power differential between social groups. We focused on adult African American/Black and Latinx (AABL) PLWH from low-SES backgrounds.

**Methods:**

Data were drawn from two studies with AABL PLWH in New York City (*N* = 59). After providing signed informed consent, participants engaged in in-depth semi-structured interviews on aspects of HIV management. Interviews were audio-recorded and professionally transcribed verbatim, and data were analyzed using directed qualitative content analysis.

**Results:**

Participants in the two studies were comparable on sociodemographic and background characteristics. They had lived with HIV for 20 years, on average (range 3–33 years). All were from low-SES backgrounds and most were African American/Black and men. Participants experienced a convergence of multiple social exclusions, harms, and stigmas, consistent with symbolic violence, which contributed to disengagement from HIV care and discontinuation of HIV medications. We organized results into five sub-themes: (1) participants were “ground down” over time by material, social, and emotional challenges and this diminished self-worth and, at times, the will to live; (2) social isolation and self-isolation, based in part on feeling devalued and dehumanized, served as stigma-avoidance strategies and mechanisms of social exclusion; (3) stigmatizing aspects of patient-provider interactions, both experienced and anticipated, along with (4) restricted autonomy in HIV care and other settings (e.g., parole) reduced engagement; and (5) poor HIV management was internalized as a personal failure. Importantly, resilience was evident throughout the five sub-themes.

**Conclusions:**

Symbolic violence is a useful framework for understanding long-term HIV management and survivorship among AABL PLWH from low-SES backgrounds. Indeed, forms of symbolic violence are internalized over time (e.g., experiencing devaluation, dehumanization, loss of self-worth, and anticipated stigma), thereby impeding successful HIV management, in part because avoiding HIV care and discontinuing HIV medications are primary coping strategies. Results have implications for interventions in community and health care settings.

## Background

Progress in controlling HIV infection is considered one of the great recent public health achievements in the United States [1]. With timely diagnosis, appropriate supports, access to a variety of HIV medications, and high levels of medication adherence, those with recently acquired HIV infections have a life expectancy equivalent to their peers not living with HIV [2]. Further, quality of life has improved among persons living with HIV (PLWH) in many respects. For example, the newer HIV antiretroviral medication regimens are simpler and more efficacious, with significantly fewer side effects, than earlier regimens [3]. Moreover, in response to improvements in state and local public health infrastructures, along with innovative and targeted outreach and treatment efforts informed by a robust research program, rates of engagement along the HIV care continuum have increased [1]. Of the estimated 1 million individuals diagnosed as living with HIV in the United States, 74% have received HIV care, 58% are retained in continuous HIV care, and 62% evidence HIV viral suppression, the ultimate goal of HIV treatment; and these rates of engagement have been improving over time [1]. In response to these HIV care and treatment advances, the lifespans of PLWH have dramatically increased in the past three decades, and a substantial proportion of PLWH in the United States have lived with HIV for 10–20 years or longer [2], spurring interest in the study of long-term HIV survivorship [4]. Yet, the benefits of these HIV care and treatment advances are not distributed equitably among PLWH in the United States. In this Background section, we first review the literature on inequities in engagement along the HIV care continuum, focusing mainly on racial/ethnic inequities, followed by a brief overview of what is known regarding the factors that drive these inequities. Following this, we summarize the modest literature on long-term HIV survivorship, introduce the concept of symbolic violence, which guides the present study, and describe its relevance for the study of managing living with HIV over the long term.

### Causes of inequity in engagement along the HIV care cascade

Racial/ethnic disparities in HIV incidence, HIV prevalence, engagement along the HIV care continuum, and health outcomes, are significant and persistent [5, 6]. First, the majority of PLWH in the United States are from African American or Black racial and/or Latinx ethic and low socio-economic status (SES) backgrounds, and, thus, over-represented compared to their proportions in the general population [7, 8]. Moreover, compared to White PLWH, African American/Black and Latinx PLWH evidence longer times between diagnosis with HIV and initiation of HIV antiretroviral therapy (ART) and between ART initiation and achieving HIV viral suppression, the ultimate goal of HIV treatment [9]. African American or Black PLWH are more likely to show suboptimal adherence to ART compared to White PLWH [10], and African American or Black and Latinx PLWH are less likely to sustain HIV viral suppression than White PLWH [11]. Yet, sustaining HIV viral suppression is critical, including as a means of preventing forward transmission of HIV to others [12]. The literature describing the factors that create these disparities along the HIV care continuum highlights a set of interconnected multi-level influences. First, low SES appears to account for at least some of these observed racial/ethnic disparities in HIV outcomes [8, 13]. Low SES creates complex competing priorities and tangible structural barriers to engagement, including unstable or low-quality housing [14, 15]. Social-level barriers include complex stigma and a lack of social support [16, 17], and, at the individual level, primary barriers include medical distrust, unemployment, and substance and mental health problems [6, 18, 19].

### Past research on long-term survivors of HIV

Buscher and Giordano [4] outline a number of major gaps in knowledge regarding the provision of care to PLWH over the long-term. They note ART adherence tends to decrease over time, but little is known about adherence patterns beyond a one- or two-year horizon. Taken as a whole, the existing research on long-term HIV survivorship has examined mainly individual-level factors that promote successful HIV management over decades, such as acceptance of one’s diagnosis, optimism, coping mechanisms, personal control, life satisfaction, focus on the self, dealing with stigma, and making meaning of HIV, including via religion/spirituality [20–24]. Further, social-level factors include social competence, social supports, human connectedness, and positive patient–professional relationships [20–23]. Structural factors that promote successful HIV management over time include medical insurance, the ability to afford medication, and access to a pharmacy [6, 25]. Conversely, as suggested above, living in poverty certainly is a critical factor driving a cascade of adverse effects on PLWH’s abilities to successfully manage HIV [6].

### The importance of understanding symbolic violence

Societies evidence various forms of violence, including the political, structural, symbolic, and every day [26]. Symbolic violence is a type of internalized, non-physical violence manifested in the power differential between social groups, and it highlights that social interaction occurs in a symbolic framework, created and maintained by society [27, 28]. Although described in the literature less commonly than other forms of violence, the framework of symbolic violence has proved useful in past public health-related research as a means of examining the ways in which social control is tacitly maintained by shared cultural practices within a given milieu without the use of force or coercion [29–32]. As an example, Winskell and colleagues [32] explored symbolic violence among sexual minorities in sub-Saharan Africa. In this context, symbolic violence took the form of internalized sexual stigma and the belief that homosexuality should be condemned and cured. Importantly, they found symbolic violence had serious adverse physical and mental health consequences, which operated through harmful stigma management strategies and minority stress [32–34]. In this context, symbolic violence took the form of homophobic rhetoric, cultural narratives, and stereotypes, which, in turn, supported and were supported by the political violence of homophobic policies, the structural violence of housing insecurity, restricted access to basic sexuality information and healthcare, and heightened vulnerability to HIV, as well as everyday heterosexist violence that sexual minority individuals faced in their communities [32]. Yet, symbolic violence and its internalized manifestations, however harmful, were found to co-occur with beliefs about acceptance of and social justice for sexual minorities [33].

This paper seeks to examine the experience of HIV management through the lens of symbolic violence to draw attention to the ways in which the multiple, marginalized social positions of stigmatized individuals and groups, including PLWH, are often experienced as natural and self-evident. This, in turn, serves to reinforce and often exacerbate various forms of social exclusion and inequality, including those related to health and health behavior. Indeed, as noted by Phelan, Link and Dovidio [35], the key functions of structural stigma, arguably one critical form of symbolic violence, are domination and oppression (keeping people “down”), norm enforcement (keeping people “in”) and disease avoidance (keeping people “away”). In addition, stigma is certainly a primary force in the lives of PLWH [36]. Thus, the concept of symbolic violence may be particularly useful in highlighting *how* internalized, anticipated, and directly experienced HIV-related and other stigmas maintain tacit social hierarchies and inequalities in ways that are not always readily apparent to those involved. We also draw on the concept of symbolic violence in an attempt to recast the experience of HIV-related stigma as more a matter of reproducing structural inequalities and social exclusions than simply of ignorance or malice on the part of individuals [37, 38]. This perspective, therefore, emphasizes the multiple layers of stigma, control, exclusion, and violence that extend beyond physicality to violations of self-worth and dignity [39, 40], and which therefore function to legitimize and preserve the various ways in which multiply stigmatized groups and individuals are frequently isolated from the rest of society.

### Questions addressed in the present study

Through the lens of symbolic violence, we are interested in how PLWH at the juncture of racial/ethnic minority status and low SES, that is, the vast majority of PLWH in the United States, experience living with a highly stigmatized chronic health condition. The research questions addressed in the present study are as follows: (1) What are the cumulative effects of living with and managing HIV over a decade or more? (2) How are forms of symbolic violence related to HIV and associated stigmas internalized and normalized, and how does this influence HIV management? (3) How do PLWH contend with symbolic violence?

## Methods

**Fig. 1:**
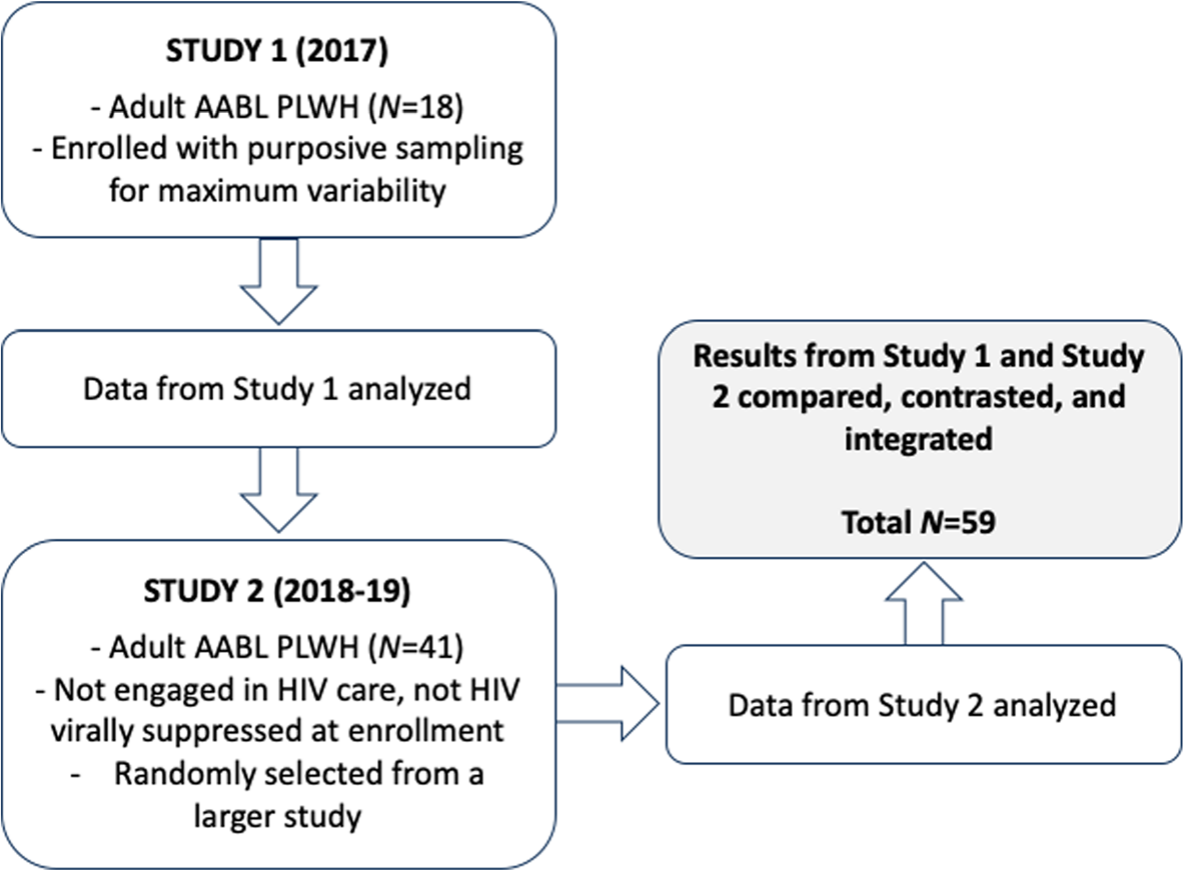
Study methods

The present study drew on individual, in-depth, semi-structured interview data from two projects conducted in New York City (NYC) with adult PLWH from African American or Black racial and Latinx ethnic and low-SES backgrounds. Individual qualitative interviews were used to allow participants to reflect in detail on their own personal experiences managing HIV and make connections among HIV management and psychosocial and structural influences where appropriate [41]. We first conducted a small exploratory study called Study 1. The aims of Study 1 (*N* = 18) were to explore barriers to and facilitators of engagement along the HIV care continuum, including long-term HIV management in its social and structural context, and identify promising new intervention approaches. Results from Study 1 indicated to researchers that the concept of symbolic violence would serve as a useful lens through which to understand aspects of contextual influences on HIV management that are under-studied to date. Results from Study 1 were encouraging, but it was modest in size. Thus, the diversity of participants’ experiences and perspectives may have been limited; that is, observational bias may have been present [42]. This raised the question of whether results were sufficiently comprehensive. To address these potential limitations and increase the depth, complexity, and validity of study findings [42], we then analyzed a second qualitative data set (*N* = 41) drawn from a larger mixed methods research study (called Study 2). The two studies are described in more detail below. Both samples included those who had lived with HIV for two decades, on average, and who also evidenced variability with respect to past and recent experiences with HIV care and ART. Results from the two studies were compared, contrasted, and integrated (Total *N* = 59; see Fig. 1). Study 1 was conducted in 2017 and approved by the Institutional Review Board at New York University, and Study 2 was conducted in 2018–2019 and approved by the Institutional Review Board at the New York University Langone School of Medicine.

### Description of the local context

The two studies were conducted in NYC, a location with a large and mature HIV epidemic of approximately 127,000 PLWH, more than 75% of whom are African-American/Black and Latinx [43]. HIV prevalence and HIV-related death rates are highly concentrated in the highest-poverty neighborhoods of NYC, which are predominantly African-American/Black and Latinx [43], highlighting the intersection of race/ethnicity and SES among PLWH, as noted above. NYC provides a large network of HIV care facilities and related support services [44], and PLWH have access to HIV care and ART at low or no cost, regardless of immigration status [44]. PLWH in NYC are typically eligible for Medicaid, a publicly funded federal insurance assistance program for low-income individuals that includes both fee-for-service and managed care plans [38, 39]. NYC has achieved higher rates of engagement along the HIV care continuum compared to national figures (e.g., 77% of all PLWH in NYC are virally suppressed [43]. Nonetheless, NYC evidences serious racial/ethnic disparities in engagement along the HIV care continuum. Racial/ethnic disparities in engagement in care, ART initiation, and viral suppression are similar to national patterns, where African American or Black and Latinx PLWH in low-SES locations show the lowest rates of engagement along the HIV care continuum compared to White PLWH in higher SES locations [8, 43].

### Recruitment

Participants for Study 1 were recruited using purposive sampling for maximum variability on key domains, including ART adherence (high/medium/low) and recent HIV viral suppression (yes/no). Further, eligibility criteria included diagnosed with HIV, age 18–65 years, and African American or Black race (referred to below as African American/Black for parsimony) or Latinx ethnicity. First, a modest number of participants (*N* = 5) were recruited from a Community Advisory Board in place for a larger study on PLWH, and these participants recruited their peers for the study. Recruitment continued until saturation was reached on core themes pertaining to new intervention approaches [45]. A total of 18 participants with diverse socio-demographic characteristics and variability in HIV histories were recruited and enrolled.

Participants for Study 2 were enrolled in a larger intervention optimization trial for PLWH with non-suppressed HIV viral load and sub-optimal engagement in HIV care using the multiphase optimization strategy (MOST) [46]. MOST is a pioneering engineering-inspired framework for testing the efficacy of individual intervention components prior to combining them into a multi-component intervention that can be tested in a randomized controlled trial [46]. Study 2 was a factorial experiment designed to test the efficacy of five separate culturally salient intervention components (patient navigation, counseling sessions, pre-adherence habit formation, peer mentorship, and support groups). Participants who declined to take ART were included throughout the larger study. Specifically, we used a fractional factorial design where participants were randomly assigned to one of 16 different intervention conditions. Each condition comprised a different combination of the five intervention components. The eligibility criteria for Study 2 included diagnosed with HIV, age 18–65 years, African American/Black or Latinx race/ethnicity, sub-optimal adherence to ART, non-suppressed HIV viral load based on a lab report, and sub-optimal engagement in HIV care. A total of 512 participants were enrolled in Study 2, described in detail elsewhere [14]. For the present study, 2–4 participants from each of the 16 intervention conditions were randomly selected to participate in semi-structured in-depth qualitative interviews 3–4 months post-enrollment. A total of 41 participants to date have participated in the qualitative interview and are included in the present study.

### Procedures and confidentiality

Procedures for the two studies were comparable: Participants were contacted by a research study staff member by telephone and asked to participate in a 60- to 90-min in-depth semi-structured interview with a trained qualitative researcher. Interviews took place in a confidential location at a research study field site. Interviews were audio-recorded and professionally transcribed verbatim. Names and other details that might identify participants personally were removed from transcripts. Participants provided signed informed consent and received compensation of $25 for their time and funds for roundtrip local transportation.

### Qualitative interview guides

In both studies, interviews followed a semi-structured interview guide collectively developed by the research team and grounded in a perspective that highlighted individual-, social-, and structural-level influences on HIV-related behavior. The interview guides included a series of questions and prompts to uncover and explore past and recent experiences living with HIV and perspectives on ART adherence and engagement in HIV primary care. Questions focused on structural-level factors, such as stable housing and access to care and ancillary services. Social-level factors included relationships with health care settings and providers (e.g., To what extent have you felt welcome and wanted at your HIV health care setting when you have been taking ART? What about when you have not been taking ART?). Individual-level domains included the participant’s experiences with ART (e.g., Have you ever taken HIV medications in the past? [IF YES] When did you start? How long did you take ART? Can you tell me what else was going on in your life at the time?). Throughout the interview process, the interview guide was updated to reflect newly emergent concepts (e.g., feeling pressured to take ART and its effects) and codes developed by the research team. Further, in Study 1 a small set of socio-demographic and background characteristics were assessed (Table 1). Study 2 included a more detailed set of quantitative indices as described below (Table 2).

**Table 1:** Sociodemographic and health characteristics of participants - Study 1 (*N* = 18)

	Mean, %
Age range in years	50–69
Male sex	56
African American or Black race (non-Latinx)	79
Latinx ethnicity	21
Low socioeconomic status	100
Receives public health insurance	100
Years living with HIV (M)	21
Range of years living with HIV	3–33
Taken ART in the past	100
Taking ART at the time of the interview with high levels of adherence	61

**Table 2:** Sociodemographic and background characteristics of participants - Study 2 (*N* = 41)

	Mean (SD) or %
Age in years	49.3 (9.05)
Age range in years	23–62
Male sex	78.0
If male, cisgender and heterosexual	62.5
If male, cisgender and sexual minority	34.4
Transgender	3.1
African American/Black race (non-Latinx)	78.0
Latinx ethnicity	19.5
In a long-term relationship	34.1
High school graduate/GED or higher	82.9
Working full-time or part-time off-the-books or on-the-books	17.1
Ran out of funds for necessities monthly or more in the past year	48.8
Food insecurity often or sometimes in past year	85.4
Stable housing (has his/her own home or apartment, including funded by government programs or benefits)	48.8
*HIV History and Health Status Indicators*	
Years living with HIV	18.5 (7.57)
Range of years living with HIV (min, max)	3.00, 33.0
Taken ART in the past	100
Number of times stopped and started ART in the past	11.3 (18.5)
Longest time on ART in the past (in months)	44.2 (64.3)
On ART with good adherence at interview	60
Participated in substance use treatment in the past	78.0
Moderate-to-high risk of alcohol problems	61.0
Moderate-to-high risk of cannabis problems	65.9
Moderate-to-high risk of other drug problems	73.2
Covered by health insurance or a health plan	95.1
Received health care for HIV in past year	95.1
Self-reported health status good or better	41.5

### Measures

Age, race/ethnicity, sex, transgender identity, sexual minority status, education, financial insecurity (how often unable to pay for necessities in the past year), food insecurity (i.e., quality/quantity/consistency of food was insufficient “often” or “sometimes” in past year), insurance status, stable housing (has his/her own home, apartment, or room that he/she rents or owns including via government rental subsidies or programs), and employment were assessed with structured instruments developed for populations in high-risk contexts [47, 48]. We assessed years living with HIV, number of times ART was stopped and started, longest amount of time on ART, use of health care in the past year, and whether ART was taken in the past 6 weeks before enrollment with a version of the HIV Cost and Services Utilization Study [49]. Self-reported health status was assessed with a single item from the RAND SF-12 measure [50]. Substance use was assessed by the World Health Organization Alcohol, Smoking and Substance Involvement Screening Test (ASSIST) [51]. Lifetime and recent substance use patterns and substance use problems across 10 substances (tobacco products, alcohol, cannabis, cocaine, amphetamine-type stimulants, sedatives and sleeping pills, hallucinogens, inhalants, opioids, and ‘other’ drugs) were assessed. Using ASSIST criteria, we describe the proportion of the sample with moderate-to-high risk of alcohol, cannabis, and other drug problems and the proportion who had engaged in substance use treatment in the past.

### Data analysis

The overall aim of the analysis was to identify, analyze, and report patterns or themes in the data set [52]. Analyses followed a directed qualitative content analysis approach [53]. This is a multi-step, iterative method of data analysis that includes immersion in the data, determining code rules and developing a codebook, performing the main analysis and inductive abstraction of main categories from codes, and then establishing links between generic and main categories or themes. Data from Study 1 and Study 2 were analyzed sequentially and then compared, contrasted, and integrated. First, after immersion in the data, a primary researcher trained in medical anthropology developed an initial code list consisting of concepts related to ART adherence, such as problems associated with low SES, a desire for good health, barriers/facilitators, and experiences, meanings, and interpretations of living with HIV. We also coded for factors related to culture and race/ethnicity (e.g., experiences of discrimination, medical distrust). Next, we conducted the main analysis of interview transcripts, as well as an inductive abstraction of main categories. Then, a second trained qualitative researcher coded a subset of the interview transcripts and met frequently with the primary data analyst. Codes and categories were further refined, and discrepancies were resolved by consensus. Findings from this initial round of coding were then presented to the larger research team, which formed an interpretive community [54]. Codes and categories were combined into larger themes and sub-themes in an iterative process and in collaboration with the interpretive community. While not explicitly included in the semi-structured qualitative interview guides, the concept of symbolic violence was selected as optimal for interpretation of and detailing the difficulties participants frequently described regarding HIV management. Symbolic violence was added to the existing codebook during Study 2, and then data from Study 1 were re-analyzed with the new codebook.

### Positionality and methodological rigor

The study was carried out by members of the research team, which was made up of people from African American, Black, Asian, Latinx, and White racial/ethnic backgrounds, diverse with respect to sexual orientation, gender, and SES. The three research team members who conducted interviews were trained in qualitative interviewing methods and research ethics. Team members had academic degrees at a master’s level or higher in fields such as social work, anthropology, public health, and psychology. All team members had substantial expertise with PLWH and several team members had expertise in race and racism. The primary data analyst (RF) was trained as a medical anthropologist and was experienced with HIV research. Positionality challenges related to sex, gender, race/ethnicity, power, health, SES, and privilege were intentionally addressed throughout the data collection process through reflection and training, which focused on the manner in which these types of issues might impact the interviewing process and data collection [55]. Methodological rigor of the analysis was maintained through an audit trail of process and analytic memos and periodic debriefing with the larger research team, which included experts in HIV care continuum issues and ART adherence, and which included PLWH [55].

## Results

### Description of participants

Participants in the two studies were comparable on key sociodemographic and HIV history characteristics, and the two samples included similar patterns of variability with respect to past and present ART use and adherence patterns, although as noted above, more detailed sociodemographic and background data were available on participants in Study 2. In both studies, participants had lived with HIV for 20 years, on average. Participants in Study 1 ranged between the ages of 50 and 69 years. More than half (56%) were men. The majority were African American/Black (79%; 15/18), and the remainder were Latinx. All were from low-SES backgrounds and received public health insurance. They had been living with HIV for 3 to 33 years, with an average of 21 years living with HIV. All had taken ART in the past, including for substantial periods of time. At the time of the present study, 61% reported taking ART with high levels of adherence in the past month (see Table 1). Those in Study 2 were 49 years old, on average, and the majority were men (78%) and African American/Black (78%). A third of the men (34.4%) in the study identified as gay, bisexual, or queer. Most participants (83%) had a high school diploma/GED or higher. Indices of extreme poverty were common: approximately half (49%) ran out of funds for necessities monthly or more in the past year and 85% reported food insecurity. Approximately half (49%) were stably housed. Participants had lived with HIV for 19 years, on average (range 3–33 years). All had taken ART in the past, including for substantial periods. At the time of the present study, 60% reported taking ART with high levels of adherence in the past month. Other background and health characteristics including substance use patterns are presented in Table 2.

### Overview of results

Overall, findings highlighted the complexity and challenges inherent in managing living with HIV over decades in the context of chronic poverty and forms of symbolic violence. On the one hand, participants understood the importance of taking ART consistently as their best chance for a long and healthy life. Yet, their years living with HIV were largely characterized by periods of institutional racism, precarity, and social isolation, during which adverse social and structural conditions were internalized as personal failings. These challenging periods were then followed by times of hard-won resilience and re-building. Over time, these repeated cycles shaped participants’ sense of self, optimism for the future, and motivation to engage in HIV care. Specifically, in addition to social isolation and chronic poverty, participants generally described their long histories of living with HIV as marked by substantial and recurring periods of emotional and material instability, including severe depression and anxiety, and cyclical or persistent substance use problems. Indeed, stopping ART or non-adherence to ART was mentioned as occurring most frequently during times of high emotional stress, depression, and heavy substance use. Participants noted that their willingness or abilities to take ART and/or to engage in HIV care were highly dependent on the degree to which they valued their own physical and emotional wellbeing within the context of a rigid social hierarchy. Moreover, even when they felt motivated enough to adhere to ART, participants reported that their efforts were continually thwarted by numerous and seemingly insurmountable structural obstacles, including housing instability, financial hardships, and/or incarceration, parole, or probation. At other times, decisions not to take ART were closely associated with an ambivalent or even traumatic relationship with both their HIV medications and the burden of HIV-related stigma, a prominent form of symbolic violence. This commonly involved participants taking deliberate or semi-deliberate breaks from their ART regimens as a means of managing these emotional correlates of living with HIV.

Thus, the concept of symbolic violence was readily apparent throughout these interviews, as participants routinely expressed experiencing multiple intersecting social stigmas, their internalizations, and their effects on health and health behavior. Among the most common manifestations of symbolic violence referenced by participants were those related to race/ethnicity, social class, gender, sexual orientation and sexuality, HIV status, substance use, and involvement with the criminal justice system. Notably, the majority of participants experienced these and other limits imposed upon them as ordinary and inevitable. Indeed, participants often considered themselves as the root cause of their own suffering, even while referencing the social and structural contexts that promoted or impeded their abilities to manage HIV effectively. For most participants, the shame and stigma attached to their HIV status began with their initial diagnosis and continued throughout their time living with HIV. However, results complicated the predominant narrative in the field and among PLWH and their social networks of the causes of HIV (namely, due to bad behavior) and “failures” with HIV management over the long term as located at the individual level. Instead, results indicated that powerful social and structural factors, including the external manifestations and internalizations of symbolic violence, shaped the individual decisions and behaviors of PLWH.

Nonetheless, participants commonly did achieve and sustain high levels of ART adherence and HIV viral load suppression for long periods of time, even in the context of symbolic violence and chronic poverty. Yet, to do so, strenuous effort and extreme resilience were required. However, participants’ periods of less optimal HIV management were generally more salient to them than times of optimal engagement along the HIV care continuum. That is, participants often blamed themselves for their perceived failures while not taking credit for their successful efforts to manage HIV. Thus, in the sections that follow we focus primarily on the factors that combined to impede ART uptake and sustained adherence, in order to give voice to participants’ experiences and perspectives and point the way to policy and health care setting changes to mitigate the effects of symbolic violence at social and structural levels to better support PLWH. Importantly, participants’ substantial strengths and resilience are embedded within this study.

**Fig. 2:**
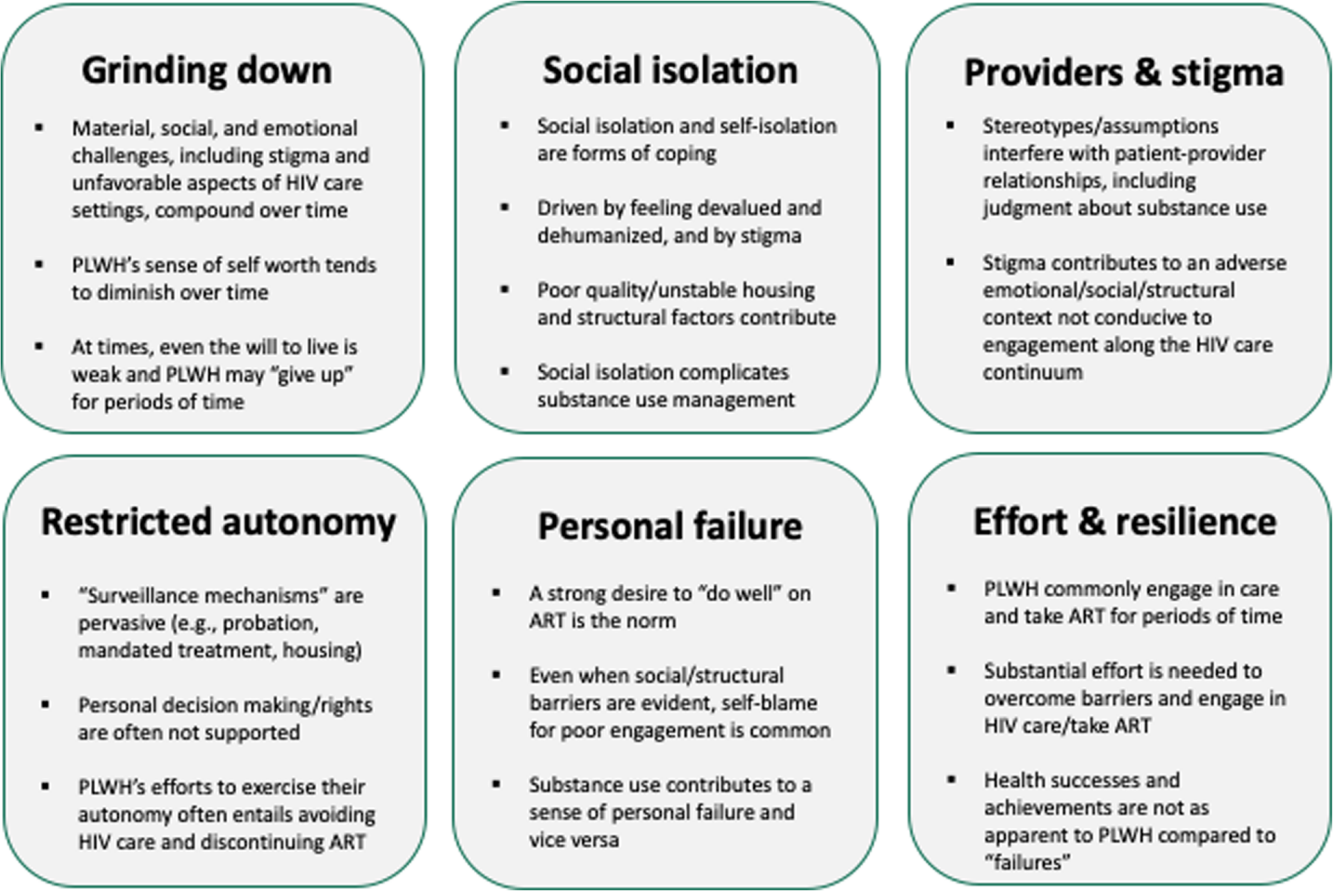
Primary themes found in the present study

**Fig. 3:**
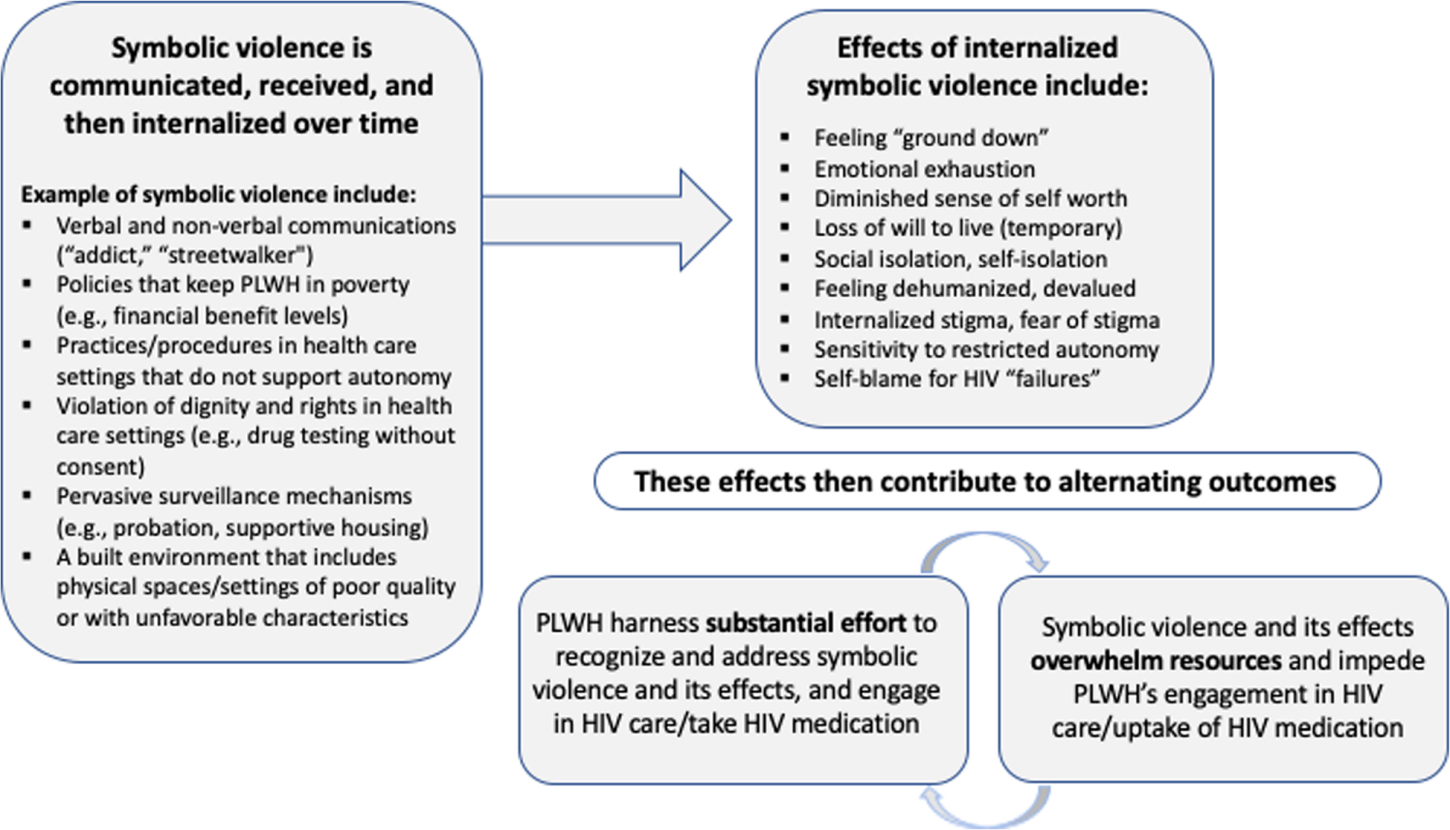
Internalized effects of symbolic violence and resultant alternating HIV-related outcomes

Overall, results were grouped into one main theme and five interrelated sub-themes. The primary theme involved symbolic violence, which produced a potent negative and counter-productive intra-psychic, emotional, and inter-personal context that served as a primary cause of nonadherence to ART. The sub-themes detailed factors that created this counter-productive context and therefore influenced the phenomenon of managing HIV health and ART over the long-term, including (1) the compounded effects of material, social, and emotional challenges, including stigma, along with life events that disrupted ART adherence, which combined over time to “grind down” participants and eventually diminish their sense of self-worth or even, at times, their will to live; (2) extreme social isolation based in part on a hyper-awareness of how they are devalued and dehumanized by society, all of which dramatically affected the emotional context within which ART adherence took place; (3) stigmatizing aspects of patient-provider interactions, both experienced and anticipated, along with (4) the experience of restricted autonomy in HIV care settings and the larger context, including mechanisms of surveillance (e.g., probation), reduced HIV care engagement and ART use; and (5) over time, the internalized experience of one’s inability to maintain ART adherence as an unmitigated personal failure, despite evidence to the contrary. The themes from the two studies were highly consistent with each other and we therefore present an integrated set of results. Names presented in the sections that follow are pseudonyms, and some identifying details have been changed to protect participants’ confidentiality. Subthemes, including the underlying theme of resilience incorporated into the other five subthemes, are summarized in Fig. 2. A summary of the predominant effects of symbolic violence, and the pathways from symbolic violence to HIV-related decisions and actions derived from the analysis, is presented in Fig. 3.

### Being ground down by compounding material, social, and emotional challenges

Participants highlighted that their relationships to HIV infection and their feelings about ART adherence were dynamic and heavily influenced by forms of symbolic violence communicated implicitly or explicitly through range of individual-, social-, and structural-level circumstances or factors. In addition to the marginalized and stigmatized social positions noted above (e.g., chronic poverty, incarceration, substance use problems), most had personal histories marked by periods of street homelessness or unstable housing (e.g., single-room-occupancy residences), chronic unemployment, domestic violence, and food insecurity. Many participants described experiencing these collective hardships as a kind of “grinding down” that resulted in feeling that one’s life or health simply was not worth the effort of the emotional, social, and practical challenges of adhering to ART. Moreover, participants commonly expressed feeling increasingly dehumanized and devalued as their years living with HIV accumulated. Importantly, taking ART served as a near-constant reminder that one was living with HIV and therefore that one would be the potential target of an array of stigmatizing behaviors on the part of strangers and loved ones alike. In many cases, PLWH appeared conscious of the effects of symbolic violence, although they did not use that term, and also showed evidence of resistance to it. Hank was a 52-year-old Black man, diagnosed with HIV 15 years ago. Hank described his awareness of the ways that symbolic violence associated with living with HIV while incarcerated affected his sense of self and behavior, as well as his drive to push back against the effects of symbolic violence by articulating its effects and seeking mental health treatment. Indeed, Hank was highly adherent to ART at the time he was interviewed, and had achieved HIV viral suppression. He noted,

Yeah, so you know during my incarceration you know with the stigma of HIV, AIDS, you know, and you have to go to what’s called pill line to get your medicine. So then you know you got people [asking] what’s wrong with him? Why you going to pill line everyday taking all these pills. You know, so you like man I’m feeling good. I ain’t going up there. That [stigma while incarcerated] and like the mental block, you know [was why I stopped ART]. It was a lot of stuff with it. So from that point on I felt like a monster who needs to be in a cage. You know and then every time I take that medicine it was like a constant reminder. All those feelings come back every time I take those pills, so that’s kind of why I’ve had a problem taking them. You know I’ve expressed that to, you know, the people [at a social service setting]. I’ve been seeking mental help.

Indeed, it is within this context that many participants experienced the evolution of their own sense of personal value and self-worth (or lack thereof), including with respect to ART adherence, HIV, and with overall health management. As Barry, a 54-year-old Black man diagnosed with HIV at the age of 24 put it,

It’s nothing you can really say to their ignorance that’ll change their mind, but I don’t think I know anybody that’s not affected by [HIV]. Either they know somebody that has it - a family member that has it. [...] I don’t see why people are cruel still with it.

Notably, the inclination to link HIV-related stigma and associated microaggressions with individually directed ignorance or cruelty, rather than with more systemic forms of social inequality as Barry did, was prevalent throughout interviews. Interestingly, however, Barry, like many participants, still expressed some degree of skepticism regarding whether individual factors were adequate in explaining the persistence and prevalence of HIV-related stigma.

Participants’ relationships to ART were also related in large measure to their initial reactions to receiving their HIV diagnoses and the difficulties they faced adapting to the new diagnosis. This was typically complicated by continuous and re-lived emotional trauma and a number of internalized social and structural stigmas. Indeed, we found a cluster of experiences around acceptance of HIV, grief and loss, continuous trauma, and stigma surrounding HIV, which created serious barriers to sustaining consistent adherence to ART. Harold, a 52-year-old Black man diagnosed with HIV while incarcerated 12 years ago, attributed these struggles directly to HIV-related stigmas:

Yeah, I struggled [with my diagnosis]. That was the hardest part about it - accepting that I was going to have to die. [...] It was hard for me. I mean, I was thinking of all the possibilities I was losing as in having kids, having a meaningful relationship and so forth. So, I lost my purpose to live. That was my reason for struggling with the whole thing [HIV care and ART adherence]. [...] But [even] now I’m stuck with this stigma of either I’m gay or I’m strung out on crack or something like that, and I get treated as such. And that’s like stuff that I’m still dealing with now with the stigma of [HIV].

Harold understood the role of discrimination in the difficulties he experienced accepting and adapting to his HIV diagnosis. This acceptance and adaptation, in turn, were necessary if he were to take HIV medication regularly. Ulysses was a 47-year-old Black man who had been living with HIV for 16 years. Similarly, he discussed his financial and mental health struggles and how these factors influenced his HIV medication adherence:

Well, I just gave up. And money was hard, so people pay for [HIV] meds, and I needed money. Sometimes I needed the money not so much for recreational use, for drugs or nothing, sometimes I just needed money for food. Or some things I needed in the house, toiletries. So I just said, hey, [I miss a month of ART], I just sell a bottle, hey. Sometimes it’s because I just give up, I’m depressed. Sometimes I lay there and – the medicine’s right there and I just don’t get up and take it. Because if my depression gets down, I don’t even have to eat. I don’t even have to drink water. I could lay there in bed for three days and don’t even have to pee. [...] I’m so depressed. Didn’t even drink any water. Just laying there.

What is notable here is that for Ulysses, his decision to sell his HIV medications was precipitated by the fact that he “gave up,” rather than vice versa. That is, for Ulysses, choosing to sell his HIV medication for basic life necessities and to voluntarily admit himself to a psychiatric ward for much-needed rest was presented as a lack of motivation to care for himself rather than the result of symbolic violence manifest in emotional, social, and structural influences. Indeed, for many participants, the decision to sell or “divert” HIV medications to purchase food, pay bills, or visit loved ones served as important factors impeding ART uptake and adherence.

Raul was a 56-year-old Latino man who was diagnosed with HIV at the age of 29 years. Raul lived in a single-room-occupancy residence, which he described “a step above living in a shelter,” but the only realistically affordable option. Similar to many other participants in the study, Raul described a combination of material and emotional pressures related to prison, parole, and his living situation that frequently resulted in what he referred to as a “fuck it attitude”:

You know, in my situation, being positive for 37-plus years, homeless, you know, it's like the worst. You know, because as soon as something doesn't fit in, you think the worst. Yeah, giving up, fuck it. F-you's and you, and fuck all this. And you run to the street. And it's not a good thing.

Importantly, this “fuck-it attitude” can be interpreted as form of resistance, resilience, and agency; it is a way that Raul has available to him to challenge structural violence, including a set of hegemonic structural inequalities. Like most participants in the present study, Raul described social service and health care settings, as well as single-room-occupancy residences (a setting where many participants resided), as dilapidated, overburdened, and over-institutionalized. Consistent with symbolic violence, these characteristics, in turn, were commonly interpreted as a near-constant reminder that participants were either not wanted or were devalued in the majority of spaces through which they traveled on a daily basis, and/or even that they were seen by larger societal forces as less than human. In fact, many participants were keenly aware of the effects of dehumanizing physical spaces. Nonetheless, Raul insisted upon establishing and maintaining his autonomy with healthcare providers despite these obstacles: “You know, they’re not going to break me. You know, they’re going to have to work with me.”

Indeed, some participants even sought to actively preempt stigmatizing interactions. Sandra, a 41-year-old Black woman who had been living with HIV for 14 years, described her strategies to manage being seen as a “pariah” as follows:

I always tell [the doctor], can you put on gloves, please? You know, because I’m still feeling that way. She’s like, girl, please.

As was the case with many other participants, Sandra’s repeated experiences of being feared and judged resulted in her pre-empting the potential experience of stigmatization (although universal precautions are intended to be de-stigmatizing since all providers are required use them for all encounters that entail patient contact). Moreover, she took ownership of it. Yet, as with many other participants, these experiences still led Sandra to avoid healthcare visits on many occasions. Indeed, the majority of participants experienced myriad inter-connected barriers to HIV management, which cumulatively led to a lengthy process that many participants described as a grinding down, as noted above, even to the point of interfering with their will to live. Put plainly, for almost all participants, forms of symbolic violence such as HIV-related and other related forms of stigma were critical aspects of a larger process that eventually led individuals to “give up” on themselves for periods of time. Moreover, these stigmas quite often served to reproduce social differences in ways that did not always involve overt forms of individual discrimination. On the other hand, participants commonly found a way out of this state of giving up on themselves, sometimes in conjunction with health care facilities and providers, and other times seemingly in spite of them.

### Social isolation and self-isolation

One of the most common strategies for managing the effects of intersecting forms of symbolic violence, specifically stigmatization and marginalization, was to avoid social interactions altogether, thereby effectively excluding themselves from much of society. Notably, this strategy included self-isolation from friends, family, and loved ones, as well as from health care and social service professionals and the larger society. Thus, in addition to feeling stigmatized in daily interactions with unfamiliar individuals, participants commonly reported similar stigmatizing experiences at home. For example, family members were known to use disposable dinnerware, refuse to share food, or spray down bathrooms or kitchens with disinfectant when participants exited. Participants often noted that they saw themselves as an outsider within their families. Further, they reported familial rejection and persistent anxiety regarding when or whether to disclose their HIV status to friends, family, and potential romantic or sexual partners over time as reasons for eventually choosing social isolation over social interaction. Ernie, a 41-year-old African American man who was diagnosed with HIV at the age of 30, described the initial shock of an HIV diagnosis as leaving him feeling “unwanted”:

That made them feel small, very small. They were inviting me over to come eat, and I wouldn't even go. Well, why don't you come? Really? Really? You're going to ask me that question? [...] Because I didn't feel like being stereotyped. I didn't feel like you running behind me every time I go to use the bathroom. [Crying] I didn't want to deal with that at all, so I just stayed away from them. I still to this day stay away from them. [...] I don't want to be talking to the ones that do know I have it, and they overhear our conversation, and then I got to deal with that [disclosure] all over again. So I just stay away, and I expand my own family by meeting people and getting close – other people that's [HIV] positive, like me.

Despite the deleterious effects of social isolation, participants commonly withdrew from much-needed social support and social interaction to avoid the emotionally punishing experiences of enacted and anticipated stigma. On the other hand, Ernie highlighted the critical role of “found family” or “family of choice,” including others living with HIV, when support from families-of-origin was lacking.

As noted above, participants frequently relied on substance use to cope with the myriad problems they faced in their everyday lives. This, in turn, often created its own set of challenges with respect to social engagement. Glen, a 47-year-old Black man who had been living with HIV for 28 years, along with co-occurring, episodic substance use problems, summed up many participants’ responses to these conditions as follows:

I always embraced venturing into the unknown, but when I started getting high it was just like I'm scared, you know, I've been doing this so long, I was getting high so long. And I secluded myself so long that it was like I was scared to go out, I was scared to succeed, I was scared to try. It was just like I was a fucking hermit, yes, and it took a minute for me to come out of that, you know.

Likewise, Rodney, was a 50-year-old Black man who learned he was HIV-positive at the age of 35 and who described self-isolation as a defense strategy that often led to “shutting down”:

I would make them [doctor’s appointments] but then I would break them because there was just so much going on. And then I’d get to drinking because I’m frustrated and [when I am] drinking I would just be like, oh, well, fuck it, go on and reschedule [the doctor’s appointment]. And there were times when I did that and made like four different appointments and wouldn’t keep them, and then after a while I was just like, okay, fuck it. [...] Like when I was just frustrated; when I just had so much of everything, problems, you know life, you know what I’m saying? Things that happen in life, you know what I’m saying? I’m the type of person, I will just pretty much shut down. When things get too hectic for me then I’ll just shut down and pretty much isolate myself. [...] That’s pretty much it, but like I said, the thing was, was when I would get annoyed, frustrated or overwhelmed with some of the stuff then it would be like I just kind of shut down and wouldn’t take [the HIV medication].

Yet, Rodney went on to highlight the cyclical nature of substance use problems and re-engagement in HIV care and ART, noting,

Then after a while I was like, no, I need to go [to HIV care]; I really need to go, you know what I’m saying, so I’d call and then make an appointment. As of now - like I said I went about a month ago - yes, I’m undetectable and all that and I’m cleared of STDs [sexually transmitted diseases]. [...] I’m more relaxed; a little more relaxed now, undetectable but I want to make sure I stay there, you know what I’m saying?

However, as the findings above illustrate, for many, avoidance of social situations was experienced as a form of self-isolation, but the structural factors driving self-isolation were all but unavoidable. For instance, many participants had been placed into supportive housing. These settings were commonly considered substandard and even potentially volatile living situations. This necessitated non-involvement with other residents, who were viewed as sources of stress, caused in large part by participants’ feeling pressured by their other residents to engage in illicit substance use. In many cases, these other residents were seen as potential perpetrators of theft or even physical violence. As Glen, introduced above, noted,

I went to [substance use] rehab in the middle of April, so I've been clean for about a month and a half, two months. Yes, so it's kind of a struggle, you know what I mean? Trying to stay clean when you got so much temptation around you. [...] Because when I was home it was just like every time the doorbell rung I would open it [but] I knew it was drugs or [someone wanting] money. You know, so I was always putting myself in harm's way.

While social engagement was vital to participants’ well-being, their fears they might accidentally disclose their HIV status to others by taking their ART in view of others exacerbated the need for self-isolation in many cases. As, Glen continued,

So I like taking my meds, it's just I forget sometimes. And then sometimes if I do remember [ … ] it was like [if somebody else] is around me and I don't want to take them, because you know, they already think you positive. But then when you pull out medication they know you're positive. But I found a way to get around that at some point [by hiding individual pills in his clothing and later taking them in private].

In fact, perhaps one of the clearest manifestations of symbolic violence among participants was not necessarily evident in the ways they experienced their everyday lives but instead, when they contrasted their typical experiences in social service and health care settings with occasional involvements in settings they experience as culturally and structurally salient. In particular, they described these latter types of settings as designed specifically to recognize and address the numerous ways this population of PLWH experiences marginalization and dehumanization. Bernard, a 54-year-old Black man who had been living with HIV for 23 years, described his positive experiences with one particular highly resourced social service and research setting as being notable in that while there he felt he was viewed in a positive light:

I've discovered that I'm really not a bad person at all. This is the overall, but I've discovered that I'm not a bad person, and I need to stop punishing myself. [...] [Before coming here] I didn't care. I didn't care.

Harold, introduced above, contrasted his positive experiences with this same service setting with those in other, far less-welcoming environments:

You know, it's like you don't feel like a pariah [there], you don't feel like nobody's scared of you because you're HIV positive. It's like people talk to you like a real person, and that matters more than anything.

Jackson, a 61-year-old African American man who was diagnosed with HIV at the age of 40, similarly described some of the reasons he believed people living in situations similar to his own feel more motivated to engage with others and to begin to care for themselves when being treated with understanding and compassion:

People come down here because they're trying to escape where they're at, and they come down here and they feel more relaxed. [Staff] say, “How are you doing? You all right?” They say good morning. Good morning. You know, and usually they're not used to that. [...] We forget that we're human. We are. We are human beings.

Indeed, participants frequently noted that their visits to this service and research setting provided far more than an opportunity to speak with staff about their personal and medical issues, and stressed the importance of being treated with dignity and respect.

### Stigmatizing aspects of patient-provider interactions

As noted above, participants consistently reported experiencing the effects of social isolation outside of health care settings. Additionally, some participants brought to many clinical interactions not only expectations of substandard medical care, but also a diminished sense of personal autonomy and a learned deference to perceived medical authorities. This, it was suggested, was often rooted in racial, gendered, or other forms of social inequity. Ernie, introduced above, related the following:

Finding the right doctor without being judged, without being discriminated against [is challenging]. I was going through several different doctors to get HIV under control, and the first thing they thought – okay, he's positive, he's Black, he had to get it from sex, and I didn't get it through sex. [...] No, I'm not a streetwalker. No, I'm not a call girl or a prostitute – nothing like that. [...] I just stopped taking medications completely.

Thus, Ernie experienced a form of verbal symbolic violence enacted through stigmatizing labelling (“prostitute,” “streetwalker”), although it was not clear whether such language was explicit, implied, or feared.

Despite commonly having numerous positive experiences with healthcare providers, participants nonetheless described acts of overt discrimination from providers, or they anticipated experiencing negative interactions with providers in the future. Harold, mentioned above, described the direct correlation between stigmatizing healthcare interactions and a reluctance to seek care at all:

Usually when you go to places like this [health care setting], people make you feel like, you know, hands off. They don't make you feel comfortable at all. It's like totally psychological, and sometimes, you know, you ain't in the mood for that shit.

Before visiting a new health care provider, participants often considered whether this new interaction would recall or even exacerbate existing traumas, including regarding their initial HIV diagnosis and the circumstances within which they believed they were infected with HIV. Sandra, introduced above, described a long series of retraumatizing healthcare experiences:

They'd just rather you die. So the care was not accessible or – to me my experience was bigoted. [...] I had a little fight left in me, so I decided to go to [another clinic] [...] They just [communicated to me], you're not important. You just like go through the mills. Like I went in there, I had a high anxiety. [...] Because everybody wanted a piece of me, is how I felt. And [I was] just overwhelmed. I'm literally crying. I broke down in tears. Lady had to give me something to calm me down.

For these and most other participants, experiences with healthcare providers were inseparable from other social interactions, and participants carried with them their experiences of dehumanization. As a result, many participants’ statements revealed the ways in which internalized and anticipated stigma sometimes resulted in expecting or even normalizing substandard medical care. Notably, many participants shared accounts of working individually with healthcare providers with whom they had overwhelmingly positive experiences, often described as “life-saving.” Nonetheless, even these participants frequently viewed the healthcare system as a whole as unwelcoming, neglectful, or even harmful, and cited this as a reason to either actively seek welcoming, non-judgmental providers, or to avoid healthcare visits altogether.

### Restricted autonomy and surveillance

For nearly all participants, feelings of distrust of and ambivalence and anxiety about health care settings were closely associated with similar emotional and social experiences in non-healthcare settings, which they generally viewed as overlapping. Participants were hyper-aware of being continuously subjected to a constellation of seemingly unrelated surveillance mechanisms, such as prison, parole, probation, court-mandated substance use treatment, supportive housing, child protective services, and, in some cases, directly observed therapy (where PLWH took ART daily in the presence of health care professionals). As a result, participants often expressed feeling their autonomy was restricted or denied with respect to personal decision making, and also noted that the harms caused to them by others were commonly minimized or dismissed entirely. As a result, participants frequently reported responding to these experiences by simultaneously expecting and recognizing a lower standard of care, but also by asserting their autonomy through an avoidance of healthcare visits or ART for periods of time. Sal, a 37-year-old Black man who had been living with HIV for three years, drew a direct line between feeling disempowered and intentionally avoiding healthcare visits:

A year ago I didn't care about being treated [for HIV]. I didn't care about taking medications. Really it was kind of because of the whole healthcare system and the frustrations I was going through as far as actually getting medicated and getting care – stuff like that. [...] They would use my medicine as a carrot. They would make me jump through hoops and do all sorts of types of things in order to get medicated, and I didn't feel that I wanted to go through that. I didn't feel that was a correct way of treating somebody with the virus. There was no compassion. We were grouped into almost a meat market of people, and everybody was just there to be treated. We were separated from other people [in an HIV clinic]. It was known that this was the [HIV] clinic, you know what I'm saying? So the whole embarrassment and stigmatism of going there to get medicated... I had decided to myself that I didn't want to go through that. [...] The whole thing was they were trying to get paid for the appointment. You know, they can bill by Medicaid [public insurance for low-income populations] for how many times I come down there for an appointment. So they were trying to get as many appointments out of me as possible, and every little thing was appointment, appointment, appointment – when I know there could've been a better or easier way.

On the other hand, even if equitable care was not expected, participants expressed frustration in the face of substandard care conditions. Indeed, many participants understood the primary function of healthcare systems, from primary care providers, HIV and other specialists, pharmacies, and incentive-driven HIV research, not as providing the best quality, convenient, confidential, and patient-centered care possible to each patient, but rather as meeting “the bottom line,” as Sal described above.

For others, interactions with various providers made it abundantly clear that participants were considered unable to make healthcare decisions for themselves. Wayne, a 52-year-old Black man who was diagnosed with HIV at the age of 28, described being tested for illicit substances without his consent, and explained how this directly led to his decision to miss appointments with his HIV care provider:

I was like, that’s fucked up. Okay. “What if I was dirty? Then what would’ve happened?” I told her. She said, “Then I would’ve handled you in a different way.” I’m like, “Handle me in a different way?” “You ain’t supposed to show no partism to a person who smokes and a person who doesn’t smoke.” You’re supposed to give them all the same treatment regardless of what they do with the medication that you give or the information that they give you. I was like, “I don’t really like the way you handle this.” So I been dealing with that. [...] And then, when I left, when I left, I missed my appointment on purpose. My next appointment, I missed it on purpose.

Similarly, Tyler explained how substance use cessation was in many cases a precondition put forth by providers for being prescribed HIV medications in the first place:

I’ve had other physicians that they’re very judgmental. You know, you’re doing drugs, you’re not going to take these meds, you’re going to sell them, so I’m not going to give you these medications. They figured this [ART] is just going to make you worse.

Yet, in contrast, Tyler’s health care provider in another setting brought a harm reduction approach to their discussions. This fostered his ability to manage HIV even when using illicit drugs, an approach consistent with autonomy support.

So over the course of, I guess years, I just became more adherent to taking the meds and I would slowly wean myself off of the other street drugs, you know.[ … ] [My doctor] was very progressive.[ … ] She was an excellent doctor, and very open and very understanding.[ … ] Not stigmatizing.[ … ] So, she was very, proactive--telling me, well, the same routine that you do your street drugs, I would like you to take these HIV meds. So, that kind of clicked in and that made me adherent. And, then, over the course of time, I just decided to reduce the harmful street drugs.

Yet, for many participants, it was more common for any sense of personal autonomy to be further undermined by an understanding that they were being deliberately taken advantage of by the healthcare system as a whole. Jackson, introduced above, described the following:

But I'll tell you one thing. I'll get tired of taking this medication because right now I'm in crisis. [...] Because it's been a battle, man. You know, let me tell you something. They experiment on a lot of us, you know, and it's sad, man. You know, using medicine, to get approval. You know, and to get in with these pharmacies. You know, come on, man, you're messing with people's lives. [...] I'm just a little fed up, that's all. You know, and you know, I keep it real with you. I [don’t] feel like going into any of these buildings [where health care is provided] because they're trying to kill me.

For Jackson, as for many participants, implicit and explicit symbolic violence in the form of stigmatization, both within and outside of clinical interactions, were commonly experienced as not only demeaning and degrading but as life-threateningly hostile. Further, they contributed to distrust of the healthcare system. Nonetheless, it is worth noting that it was within discussions of autonomy (or a perceived lack thereof) that the effects of symbolic violence were readily visible, as participants continually struggled to locate themselves positively within the healthcare system as a whole.

### The sense of personal failure if not on ART

Despite experiencing and recognizing all of the emotional, social, and material challenges enumerated above, participants who had achieved HIV viral suppression or who had successfully adhered to ART nonetheless often retroactively interpreted past ART periods of non-adherence as the result of personal shortcomings. That is, for many participants, successfully attending HIV care appointments and adhering to ART in the present often served as a powerful reminder of what many perceived as personal failings in the past. Despite facing myriad obstacles related to meeting their daily needs, participants nonetheless continuously expressed a desire to “do well” with respect to their health. Indeed, for nearly all participants, and consistent with health care providers’ views, biomarkers such as high CD4-cell counts and low HIV viral load levels served as markers of success. However, many participants’ self-narratives suggested that, although they understood the context within which they were expected to adhere to ART as prohibitive or at times even overtly hostile, they nonetheless frequently viewed themselves as partially to blame for their lapses in HIV care engagement and/or ART adherence. For instance, many participants noted that they felt that only once one achieved a certain CD4-cell count or HIV viral load level would they deserve equitable treatment in society. As Sandra, introduced above, noted,

I felt like people were judging me or, you know, they don't know my story the way they act towards you, especially if you're coming in with such a high viral load. They look at you like you're careless. You don't know my story, you don't know what I've been going through. You don’t know that I have private insurance and I got kicked off my insurance plan because of my, you know, my illness. You don't know the background of why I'm not able to get my meds.

Similarly, Mona, a 33-year-old Latina woman who was diagnosed with HIV at the age of 23, faced deeply interconnected barriers to achieving these favorable HIV outcomes:

Of course, yes, I should be taking my meds. You know, I want to take my meds. I know and hear the importance of taking your meds. It’s already proven that without meds I become very ill. [...] Right, but the biggest problem right now is money, right. And sometimes you say you just don’t feel like taking [the meds] … Honestly, I would just want my CD4 to be in the normal range, or at least higher than 28 [a very low CD4 cell count] so I could feel good and feel better. And maybe I think when you feel better physically you think better mentally. So you know, I [would] feel like I’m a regular person. When I’m sick and feeling not so well I’m a little depressed, you know, and I go through my little shit. But when everything seems okay you want to take your meds, you want to do good.

For Mona, feeling “a little depressed” and unlike “a regular person” often spiraled quickly into a situation wherein she ended up feeling “dead inside” and missing doses or selling/diverting her medications altogether, which she noted repeatedly was often her only option for survival: “Yes, honestly (when) it’s not difficult … I don’t have to sell them.” As is the case with many participants, Mona saw depression, stigmatization, and financial necessity as deeply interconnected, if not indistinguishable. Again, within these narratives, the unwillingness or inability to regularly take HIV medications was sometimes understood as a personal failure rather than a failure of a larger system to provide appropriate supports, including an understanding of the symbolic violence and structural barriers that characterized so many aspects of their lives. Mona, introduced above, described her situation as follows:

Nobody wants to be a bum or a drug addict, and it just doesn't happen, life happens. You know, sometimes when you don't have a friend you don't know how to cope. And you turn to drugs, and you get fucked up physically, you know, you just change who you are. I felt like that was happening to me too. [...] I just want to enjoy life for what it is. I don't want to be rich. I just want normal. I just want furniture in my living room. I want everything a normal mom wants. I don't want nothing more. I don't want nothing less. That's it. I just think I compromise too much.

Like Mona, nearly all participants described “normality” as an all but untenable goal in the past, present, and/or future, and viewed themselves as largely responsible for having not achieved that goal, an internalization of symbolic violence.

## Discussion

Racial/ethnic disparities in HIV incidence, prevalence, morbidity, and mortality in the United States are well documented [7]. Yet, relatively little is known about the experience of living with and managing HIV over a decade or more, including among those who experience these disparities. Societies evidence various forms of both direct and indirect violence (i.e., political, structural, symbolic, and every day) [26]. However, symbolic violence is under-studied compared to these other forms, including in the field of HIV. The present study took a qualitative approach to address this gap in the literature. We sought to better understand the role of symbolic violence in creating and maintaining racial/ethnic and other disparities in HIV, and the mechanisms by which it does so, from the perspectives of African American/Black and Latinx PLWH from low-SES backgrounds in an urban environment who have lived with HIV for 20 years, on average. We also attended to ways this subpopulation of PLWH often recognizes and resists symbolic violence, and we suggest potentially modifiable aspects of, and remedies to, symbolic violence and its correlates and effects, as we detail below.

### The potency and omnipresence of symbolic violence in the lives of PLWH

Symbolic violence is a useful organizing concept for understanding the experience of long-term HIV survivorship for African American/Black and Latinx PLWH. In particular, symbolic violence is a valuable framework for interpreting participants’ complex relationships with exclusion, self-perception, physical and psychological harms, and HIV-related and other forms of stigma. Moreover, it permits a description of participants’ understandings of the cumulative effects of social and structural contextual factors on the sense of self, social life, health care decisions, and experiences with health care and social service settings and providers. Although symbolic violence does not begin when an individual receives an HIV diagnosis, clearly HIV confers forms of symbolic violence highly specific to living with HIV, along with forms of symbolic violence that affect other marginalized populations. We found symbolic violence is enacted both explicitly and implicitly, including through verbal and nonverbal communication directed toward PLWH, through terms such as “addict,” “prostitute,” “streetwalker,” “gay,” and “strung out on crack.” Even the highly charged term “AIDS,” the use of which has recently been discouraged by public health experts [56], is often experienced as a symbolically violent microaggression, in that it can serve to remind PLWH of their marginal position in society. Further, health care and social service policies, such as financial entitlement benefit levels that are not sufficient to prevent extreme poverty and food insecurity, commonly act in ways that are symbolically violent. Symbolic violence can also manifest in professional relationships, such as when health care providers are experienced as dismissive or not supportive of personal autonomy, or even as violating of dignity and human rights (e.g., believing one is being drug tested without prior consent). The larger society conveys symbolic violence to PLWH through mechanisms of control and surveillance such as mandated substance use treatment, and probation and parole. Moreover, the built environment is often symbolically violent, such as when PLWH in low-SES environments experience health care facilities and supportive housing placements as sub-standard compared to those available to their peers in the higher socioeconomic statuses, and when HIV clinics are conspicuous and located separately from other facilities, which serves to reinforce stigma and make it challenging to keep one’s HIV status confidential. Thus, symbolic violence is certainly pervasive in the lives of African American/Black and Latinx PLWH.

### Internalization of symbolic violence

We found that symbolic violence is commonly internalized over time, which creates a counter-productive intra-psychic, emotional, and inter-personal context that, results suggest, contributes to poor engagement along the HIV care continuum, as shown in Fig. 3. This, in turn, reinforces and perpetuates various intersecting forms of social exclusion and marginalization, including those based on race/‌ethnicity, gender, social class, sexual orientation, substance use, and HIV status. For example, among participants in the present study, years of substandard treatment, neglect, hassles, challenges, and, at times, outright discrimination commonly result in a diminished sense of self and capacity for change. Moreover, over time, symbolic violence and its manifestations commonly “grind down” PLWH and eventually diminish their sense of self-worth and, even their will to live in some cases. PLWH then typically view their challenges with engagement along the HIV care continuum as a result of personal deficiencies, rather than as the result of these serious structural and social barriers. Indeed, over half of participants in the present study were taking ART with high levels of adherence at the time the qualitative interviews were conducted. Nonetheless, participants’ narratives rarely highlight that major achievement, suggesting that participants’ perceived failures tend to be more readily apparent to them than their successes. Nonetheless, results from this study overall underscore that participants’ responses to internalized symbolic violence and stigmas are more strongly rooted in these larger social structures than in individual attitudes.

### Contending with symbolic violence in the health care system

PLWH experience symbolic violence in the HIV care system, which is perhaps not surprising given that this system is located within the larger societal context. Indeed, navigating the highly complex landscape of HIV-related healthcare is exceedingly difficult for PLWH, in large measure due to symbolic violence. Many HIV care settings provide comprehensive care and care coordination [57], and the field of primary care overall is moving toward integrated models of care [58] and providing liaisons, navigators, coordinators, and even concierges to assist patients with managing care [59]. Yet for participants in the present study, even moderate success managing the health care system required effort, shrewdness, and sophistication on their parts. Moreover, engagement in HIV services necessitated a willingness to consider that seeking care had the potential to cause harm to one’s emotional and physical health (e.g., when autonomy was not supported, or PLWH felt they were treated like an “addict” or “prostitute”). Moreover, participants highlighted an ancillary issue: their desire to access care frequently requires them to ascertain the degree to which their needs are actually being met by particular providers, and then decide which providers to engage with based on this assessment. These specific challenges add complexity to engagement in HIV care and contribute to the internalization of symbolic violence over time, but are not well-addressed by existing care coordination and navigation models.

Substance use is a common point of friction between participants and healthcare systems providers. PLWH who use substances or have done so in the past commonly experience, or anticipate they will experience, judgment from healthcare providers, which, they fear, may result in denial of care [60, 61]. Indeed, these stigmatizing attitudes toward and substandard treatment of PLWH who use or are perceived to use substances is another form of symbolic violence. In fact, there is a substantial literature on symbolic violence and its effects on persons who use substances, as they are another population that is considered socially and culturally controlled [31]. However, HIV health care providers may not always have the skills to engage patients around substance use concerns, particularly in the context of a short health care encounter [60, 61]. Nonetheless, substance use is an important juncture for PLWH where symbolic violence is communicated and potentially internalized, and which contributes to discontinuation of HIV care and ART.

Thus, particularly in low-SES environments, it is difficult for PLWH to access social service and medical settings that seek to counteract and/or not perpetuate symbolic violence by supporting participants’ dignity and autonomy, combatting dehumanization, and actively and explicitly acknowledging during the course of care the important social and structural factors that contribute to a lack of engagement along the HIV care continuum. Yet, when PLWH do access such settings, the emotional benefits are apparent and are often followed by improvement in HIV-related health behaviors. It must be noted, however, that most participants report having numerous positive and even life-changing and life-saving experiences with their own personal healthcare providers. Indeed, the substantial rates of engagement along the HIV care continuum locally and nationally highlight that for most PLWH, the HIV care system meets and often even exceeds basic needs. However, for the substantial proportion of PLWH who are poorly engaged along the HIV care continuum, experiences with the healthcare system as a whole, often combined with dehumanizing experiences within social service agencies, supportive housing, and the criminal justice system, commonly coincide with healthcare systems in such a way that these systems are collectively experienced as indifferent or hostile.

### The present study advances the literature on stigma

Study results are consistent with past research on stigma and its contribution to social isolation [62]. Results from the present study advance the literature by exploring PLWH’s perspectives on mechanisms through which factors such as perceived stigmatization from friends and family, unfavorable housing environments, and anticipated or experienced adverse conditions in other social settings can lead to extreme social isolation. Moreover, this study illustrates the ways these phenomena are compounded when PLWH understand extreme forms of social isolation as an individual failure, rather than as a result of societal-level stigma and substandard supports available to those living in poverty. Indeed, social isolation is a very serious public health concern related to elevated morbidity and mortality rates [﻿63﻿]. Overall, results demonstrate that HIV and other related stigmas are indeed frequently seen as manifested in individual social interactions, including patient-provider interactions [64–66]. Yet, perhaps one of the most pernicious aspects of both external and internal stigma is its ability to maintain structural inequities such as unequal access to and differential treatment by the healthcare system, implicit exclusion from social spaces, and lack of autonomy in personal decision-making, in ways that were often hidden from plain sight. Of serious concern, PLWH commonly withdraw from HIV care altogether and discontinue ART as a means of exercising self-determination and coping with the negative affect inherent in these strained encounters.

### Counteracting the effects of symbolic violence

Although symbolic violence is largely unnoticed and communicated as a “normal” aspect of society, we found PLWH often recognize symbolic violence and its effects. Moreover, the present study uncovered a wide array of strategies that this subpopulation of PLWH develops to cope with its many manifestations. Pro-social and health-enhancing strategies include anticipating and recognizing the sources and effects of symbolic violence and seeking mental health services or substance use treatment, creating a “found family” of other PLWH, and deciding to re-engage in HIV care. Yet, clearly other approaches PLWH use to manage symbolic violence are less productive in the long term, such as self-isolation, withdrawing from HIV care, and discontinuing HIV medications, as we detail throughout the study. Yet, disengagement from HIV care and/or ART are often best understood as common conscious applications of personal autonomy (e.g. “I had a little fight left in me” and “they’re not going to break me”).

### Implications for policy, practice, and future research

As Williams and Jackson [﻿67﻿] note, efforts are needed to identify and maximize health-enhancing resources that may reduce some of the negative effects of psychosocial factors on health. Consistent with the standpoint taken in the present study, they outline how health and health disparities are embedded in larger historical, geographic, sociocultural, economic, and political contexts. Thus, changes in a broad range of public policies, including those outside traditional health policy, are likely to be central to effectively addressing racial/ethnic inequities such as those in HIV [﻿67﻿]. In Table 3 we present practical recommendations for changes in policy and in medical and social service practice settings that emerged from the present study, grounded in the ways we found symbolic violence is communicated to African American/Black and Latinx PLWH, how they internalize it, and its numerous adverse effects. Taken together, these recommendations have potential to mitigate the effects of symbolic violence and provide an environment conducive to effective HIV management by promoting the interests of “non-dominant” groups as well as reducing distinctions and hierarchies of ranking between dominant and non-dominant groups, thereby challenging the status quo [﻿39﻿]. Further, more research is needed on the reasons why and the mechanisms by which individuals are dominated and oppressed (kept “down”), enforced by norms (kept “in”) and avoided (kept “away”) [﻿35﻿]. Many of these frameworks and initiatives in Table 3 are being implemented in some settings, including NYC, which has made recent substantial progress engaging PLWH along the HIV care continuum, as described above. Nonetheless, approximately 30% of PLWH in New York City are not virally suppressed, mainly African American/Black and Latinx PLWH from low-SES backgrounds, and, as noted above, once achieved, viral suppression may not be sustained. To sustain gains made and improve engagement along the continuum, enhancements in policy and practice and poverty and stigma reduction efforts are needed even in resource-rich settings with a large and mature HIV epidemic such as NYC. Notably, the present study highlights the need for research on the population of those living with HIV over decades. As Buscher and Giordano [﻿4﻿] have noted, currently there is no unifying concept for studying this population of long-term HIV survivors (they suggest the term “HIV survivorship research,” which we use throughout this paper), and interest is scattered throughout different fields and results are presented in various clinical, behavioral, public health, and health services forums.

**Table 3:** Recommendations that emerged from the present study

Overall lesson learned	Specific recommendations
Poverty is a fundamental cause of HIV-related health and other social inequities	▪ Provide universal basic income▪ Reduce barriers that prevent eligible individuals from accessing benefits [68]▪ Increase entitlement levels, as current sub-poverty benefit levels ensure continued hardship [68]▪ Entitlements and health benefits are generally subject to strict low-income guidelines, which precludes employment for many PLWH who need to retain benefits. Changing these policies could increase employment rates [69]▪ Provide job training programs in health care and social service settings, as employment can increase knowledge, money, power, prestige, and beneficial social connections and reduce the fundamental causes of disparities [70]
Stigma is a fundamental cause of HIV inequities	▪ Address community-level stigma within its broader structural context (e.g., CHHANGE study) [71]▪ Implement symbolic violence and stigma-reduction training and intervention efforts at the levels of health care systems, providers, and PLWH
Substance use is chronic and recurring	▪ Provide interventions to health care settings to reduce substance use-related stigma▪ Locate specialized retention clinics within HIV clinics to support persons who use substances [72]▪ Ground services in harm reduction, emphasizing support for individual autonomy and decisions▪ Locate evidence-based substance use treatment in HIV care settings
Housing is often precarious, coercive, and of poor quality	▪ Provide high-quality and stable housing to reduce dehumanization, social isolation, and exposure to others with substance use problems [73]
The physical and social characteristics of health care/social service settings can be experienced as dehumanizing	▪ Design health care settings to be open, transparent, and inclusive, consistent with the concepts of spatial and placial justice [74]
Aspects of health care/social service encounters can support HIV management but may be lacking in poorly-resourced settings	▪ Implement approaches in clinical settings that support PLWH’s autonomy to better foster engagement and decision making▪ Implement and train providers in stigma-reducing approaches that include a non-judgmental approach to possible ART non-persistence, substance use, and other aspects of PLWH’s lives that may be stigmatizing▪ Develop and implement practices that combat dehumanization and devaluation▪ Integrate motivational interviewing [75], strengths-based [76], and person-centered care approaches into services [77] because they have a strong evidence base, foster engagement, and support PLWH’s resilience and autonomy▪ PLWH miss HIV care appointments as one strategy to manage HIV over the long-term, but taking PLWH off patient rosters in response to missed visits creates barriers to their accessing HIV care
Negative emotions impede engagement, but are less commonly the focus of care/services than other aspects	▪ Implement interventions in clinical and social service settings that attend to emotional factors, along with those that focus on cognitions and behavioral skills [78, 79].▪ Acknowledge and address fear and distrust common among African American/Black and Latinx PLWH [78, 80–82]▪ Acknowledge and address other negative affective states and include programming to help participants manage negative emotions [78, 80–82]
Continuous traumatic stress is endemic and chronic	▪ Provide services to address the sequelae of a traumatic HIV diagnosis experience, and the often non-linear and challenging process of accepting and adapting to the diagnosis [24]▪ Train providers in trauma-informed care and integrate trauma-informed care in clinical and social service practice [83]
PLWH often prioritize individual “failings” despite myriad accomplishments	▪ Help staff and PLWH understand and acknowledge social and structural drivers of poor HIV management, called structural competence [84]▪ Highlight resilience and strengths in clinical encounters

### Limitations

This study has a number of methodological strengths, including the use of data from two separate studies and a methodological approach designed to improve transparency, rigor, trustworthiness, and validity [42, 55, 85]. The present study also has limitations. One potential limitation is the purposive sampling method for Study 1. Second, Study 2 enrolled only the subset of the larger population of PLWH with barriers to engagement along the HIV care continuum and non-suppressed HIV viral load at the time they joined the study (although 60% were taking ART at the time of the interview). Further, participants were 50 years old, on average. These sampling factors may limit our ability to generalize results to the population of African American/Black and Latinx PLWH as a whole, including younger PLWH. Yet, purposive sampling and focus on this subpopulation are consistent with the goals of qualitative research, which aims for depth rather than breadth. Moreover, retrospective accounts such as these may be subject to primacy and recency effects, and other cognitive and memory biases [86–88]. On the other hand, recall of life events using qualitative methods has validity [89]. Because all participants in the study were African American/Black and Latinx, the interview guides used did not include explicit questions regarding the potential role of race/ethnicity in long-term HIV survivorship, which may have limited study findings in this domain. Nonetheless, as described above, participants did introduce experiences associated with race/ethnicity. Further, we had less quantitative data on the sociodemographic and background characteristics of participants in Study 1 compared to Study 2. Nonetheless, findings from Study 1 were valuable, and we have included them in the present study. Another limitation is that participants in Study 2 were engaged in a larger intervention optimization trial to test intervention components. Although we interviewed participants early in their time in Study 2, these intervention activities may have shaped their perceptions of their HIV-related decisions and behaviors. On the other hand, their retrospective reflections on decades living with HIV were highly consistent with those in Study 1, which supports the validity of this study. Although the sample size was substantial for a qualitative examination of this nature, it did not allow us to examine age, sex, or racial/ethnic differences in detail, a gap that future studies on this topic can address. Last, the present study did not include respondent triangulation, such as interviews with health care providers or other stakeholders. Indeed, such triangulation would have allowed us to examine the experience of HIV management from different perspectives and thereby validate results through cross-verification [90].

## Conclusion

Despite major recent advances in HIV treatment and improvements in engagement along the HIV care continuum leading to reduced morbidity and mortality rates for PLWH, African American/Black and Latinx PLWH experience more barriers to engagement, as well as poorer health outcomes, compared to their White peers. Symbolic violence is a useful framework for uncovering and exploring the understanding, meaning, and experiences of African American/Black and Latinx PLWH from low-SES backgrounds who have been diagnosed with HIV 20 years ago, on average; the structural, social, and intrapsychic effects of a long-term HIV survivorship; as well as identifying a number of critical factors that promote or impede successful management of HIV, many of which are addressable. Study findings have potential implications for interventions in community and health care settings.

## Data Availability

Data are available upon request from the corresponding author.

## Abbreviations

AABL: African American/Black and Latinx
ART: Antiretroviral therapy
CD4: Cluster of differentiation 4 (also called t-cells)
HIV: Human immunodeficiency virus
MOST: Multiphase optimization strategy
NYC: New York City
PLWH: Persons living with HIV
SES: Socioeconomic status
